# P-252. Culture-based Viability PCR: Strategies to Harness its Sensitivity and Minimize False Positives

**DOI:** 10.1093/ofid/ofae631.456

**Published:** 2025-01-29

**Authors:** Erin Duane, Ayanna Sharma, Evan Oldenburg, Aaron Barrett, Guerbine Fils-Aime, Amanda M Graves, Deverick J Anderson, Bobby G Warren

**Affiliations:** Duke School of Medicine, Durham, North Carolina; Duke University, Greensboro, North Carolina; Duke University, Greensboro, North Carolina; Duke Health, Cary, North Carolina; Duke School of Medicine, Durham, North Carolina; Duke University School of Medicine Duke Center for Antimicrobial Stewardship and Infection Prevention, Durham, North Carolina; Duke Center for Antimicrobial Stewardship and Infection Prevention, Durham, NC; Duke University School of Medicine, Hillsborough, North Carolina

## Abstract

**Background:**

qPCR is a highly sensitive method for identifying the presence of potential pathogens. However, its utility as a tool to detect environmental contamination is limited by its inability to differentiate between viable and non-viable target cells.

**Table 1.**
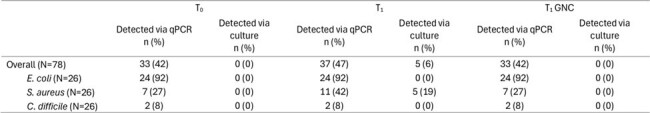
Detection of target organisms via qPCR and Culture

**Methods:**

We completed a prospective microbiological analysis of patient bed footboard samples at a tertiary care center using foam sponges and the stomacher method to process samples. Target species included *E. coli* (EC), *S. aureus* (SA), and *C. difficile* (CD).

Sponge homogenates were split into three paths: 1) T_0_: 500uL was added to 4.5ml of species-specific (SS) broth; 500uL of the resulting mixture underwent DNA extraction and qPCR with SS primers, 2) T_1_: 500uL was added to 4.5mL of SS broth, and 3) Growth negative control (GNC): 500uL was added to 4.5mL of 8.25% sodium hypochlorite, incubated for 10 minutes, centrifuged for 15 minutes at 3100 RPM, then decanted and added to 5mL of SS broth after 2 PBS washes. T_1_ and GNC samples were then incubated at SS conditions (24 hours at 37C for *EC* and *SA*, and 48 hours for *CD*). After incubation, 500uL from T_1_ and GNC samples underwent DNA extraction and qPCR. All samples were also cultured on SS agar. A sample was considered viable for each species if 1) it was detected at T_0,_ and the CT decreased by at least 1.0 at T_1_ compared to GNC or 2) it was undetected at T_0_, detected at T_1_, and undetected for GNC, or 3) grew on standard culture agar.

**Table 2:** Viability of target organisms via qPCR and Culture

**Figure:**
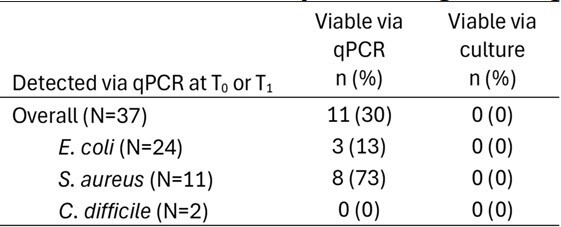

**Results:**

468 samples from 26 patient rooms were analyzed, including 156 for each species. Of the 26 original samples, 24 (92%), 11 (42%), and 2 (8%), had detectable levels of *EC*, *SA,* and *CD* via qPCR at T_0_ or T_1_, respectively, and could be assessed for viability. Of those, 3 (13%), 8 (73%), and 0 (0%) contained viable cells of *EC*, *SA* and, *CD* via qPCR, respectively. Notably, 5 (19%) of *SA* samples were culturable at T_1,_ indicating broth enrichment enhances culture sensitivity; however, all were determined viable via qPCR as well.

**Conclusion:**

Culture-based viability PCR outperformed traditional culture methods in detecting viable pathogens with improved specificity compared to qPCR, highlighting its potential as a tool for assessing environmental contamination. Further large-scale studies are needed to confirm these results across different species.

**Disclosures:**

**All Authors**: No reported disclosures

